# Contralateral R1 response in blink reflex in patients with amyotrophic lateral sclerosis

**DOI:** 10.1016/j.cnp.2025.02.005

**Published:** 2025-02-22

**Authors:** Julian Theuriet, Adrien Bohic, Maxime Bonjour, Emilien Bernard, Florent Cluse, Juliette Svahn, Laurent Jomir, Anne-Evelyne Vallet, Marion Demia, Lucie Roux, Ioana Cristina Bârsan, Léa Alves, Matthias Dion, Lionel Meens, Martin Moussy, Françoise Bouhour, Yann Péréon, Antoine Pegat

**Affiliations:** a Service d’ENMG et de Pathologies Neuromusculaires, Centre de Référence des Maladies Neuromusculaires PACA-Réunion-Rhône-Alpes, Hôpital Neurologique Pierre Wertheimer, Hospices Civils de Lyon, Groupement Est, 69500 Bron, France; b Centre de Référence SLA et Autres Maladies du Neurone Moteur, Hôpital Neurologique Pierre Wertheimer, Hospices Civils de Lyon, Groupement Est, 69500 Bron, France; c Service de Biostatistique, Pôle Santé Publique, Hospices Civils de Lyon, Lyon 69002, France; d Laboratoire de Biométrie et Biologie Evolutive UMR 5558, 69100 Villeurbanne, France; e Centre de Référence des Maladies Neuromusculaires AOC, Euro-NMD, Filnemus, CHU de Nantes, Hôtel-Dieu, 44000 Nantes, France

**Keywords:** Blink reflex, Amyotrophic lateral sclerosis

## Abstract

**Objective:**

This study aimed to compare the frequency of blink reflex’s contralateral R1 responses (R1′) between patients with amyotrophic lateral sclerosis (ALS), non-ALS motor deficit patients, and healthy volunteers.

**Methods:**

A total of 120 participants were prospectively recruited: 40 with ALS, 40 with a non-ALS motor deficit, and 40 healthy volunteers. Blink reflexes were recorded from orbicularis oculi muscles following supraorbital nerve stimulation.

**Results:**

R1′ was more frequent in the ALS group (42.5 %) compared to healthy volunteers (12.5 %, p = 0.00588), and compared to non-ALS patients (7.5 %, p = 0.000789). Bilateral R1′ was observed only in ALS patients (22.5 %). No clinically significant difference was found in the latencies or amplitudes of the R1, R2, or R1′ responses among groups. R1′ was more frequent in ALS patients with pseudobulbar affect (71.4 %) compared to those without (36.4 %).

**Conclusions:**

The higher frequency of R1′ in ALS highlights its potential role in distinguishing ALS from other motor disorders. Its sensitivity was low, but bilateral R1′ was specific to ALS. The higher frequency of R1′ among ALS patients with pseudobulbar affect potentially reflects corticobulbar neuron degeneration.

**Significance:**

The R1′, especially when bilateral, could serve as an additional diagnostic biomarker for ALS, although its clinical relevance should be considered within the broader diagnostic context.

## Highlights

• Contralateral R1 responses (R1′) in the blink reflex are more frequent in ALS patients than non-ALS and healthy volunteers.
• Bilateral R1′ was specific to ALS, observed in a quarter of the ALS group and absent in non-ALS and healthy volunteers.
• R1′ in ALS may serve as a diagnostic biomarker though it lacks sensitivity and must be interpreted in the clinical context.

## 1 Introduction

Amyotrophic lateral sclerosis (ALS) is a neurodegenerative disorder characterized by the progressive loss of upper and lower motor neurons (Feldman et al., 2022, Van Es et al., 2017). It clinically manifests as a rapidly progressive motor weakness that develops within weeks or months. It can affect limb, bulbar, and/or respiratory muscles, progressively leading to death by respiratory insufficiency (Feldman et al., 2022, Van Es et al., 2017). Although no cure is currently available, early diagnosis is important for correctly monitoring patients' respiratory function, detecting the genetic form to properly inform patients and their families, and allowing access to clinical trials (Feldman et al., 2022, Van Es et al., 2017). Electrodiagnostic (EDX) studies are part of the diagnostic criteria of the disease, are necessary to rule out other diagnoses, and can shed light on the pathophysiology underlining this degenerative disorder (Goutman et al., 2022, Shefner et al., 2020). Nerve conduction studies can show reduced amplitude compound muscle action potentials (CMAP), while needle electromyography (EMG) can reveal abnormal resting activities such as fibrillations and/or fasciculations, and polyphasic, enlarged, and high-amplitude motor unit action potentials (MUAP) with reduced recruitment during contraction (Goutman et al., 2022).

The blink reflex investigates the trigeminal-facial pathway (Kofler et al., 2024, Pearce, 2008). Electrical stimulation of the supraorbital nerve results in two responses: an early component (R1) ipsilateral to the stimulus, transmitted through an oligosynaptic arc in the pons, and a bilateral late component (R2), which follows a long polysynaptic pathway traveling through the spinal nucleus of the trigeminal nerve (Kofler et al., 2024, Pearce, 2008). In another disease affecting the upper motor neurons, human T-lymphotropic virus 1 (HTLV1)-associated spastic paraparesis, an early contralateral R1 response (hereafter referred to as R1′) in the blink reflex has frequently been observed (Leon-S et al., 1995). This response was attributed to the lack of some inhibitory input at the interneuronal level resulting in facial nucleus hyperexcitability (Leon-S et al., 1995).

Thus, it can be hypothesized that a R1′ could be an interesting electrophysiological marker of ALS. The main objective of the study was therefore to compare the frequency of R1′ in ALS patients with that in healthy volunteers and patients presenting motor weakness and/or fasciculations from another cause.

## 2 Methods

### 2.1 Study participants

All participants were prospectively recruited from the ALS reference center and the neuromuscular reference center of Lyon, France. Consecutive patients who were referred for motor weakness involving bulbar or limb muscles and/or fasciculations for an EDX study were included. Patients were included from February 2024 to August 2024. They were classified into two groups: i) the ALS group if they fulfilled the Gold Coast criteria at the end of the EDX study, and ii) the non-ALS group if they did not fulfill the Gold Coast criteria (Shefner et al., 2020). Patients with a history of facial nerve or central nervous system involvement were excluded from this study to avoid interference with the blink reflex results. Healthy volunteers, who were healthcare professionals working in the authors’ hospital, were also included and served as controls. The study approval (ID RCB 2024-A00016-41) was granted by the competent ethics committee (*Comité de protection des personnes Ile-de-France*), on 11 February 2024. The study protocol was registered on the ClinicalTrials.gov website (NCT06206629). All patients were given an information letter and gave their oral consent to participate in the study, in accordance with legislation in place at the time of the study.

### 2.2 Clinical evaluation

The clinical evaluation of the patients was performed on the day of the EDX study and included sex, age at inclusion, disease duration, onset site, presence of bulbar and spinal symptoms, revised ALS functional rating scale (ALSFRS-R), Centre for Neurologic Study-Lability Scale (CNS-LS), and the Montreal Cognitive Assessment (MoCA) score if cognitive impairment was suspected. A pseudobulbar affect was defined by a CNS-LS score > 12 (Moore et al., 1997). Cognitive impairment was considered if the MoCA score was 25 or lower. Spinal involvement was defined by motor weakness in a limb, bulbar involvement by the presence of dysarthria and/or dysphonia and/or swallowing disturbance, and respiratory involvement by dyspnea and orthopnea. The presence of pyramidal syndrome was defined by the presence of brisk reflexes and/or a Babinski sign and/or a Hoffman sign and/or epileptoid tremor of the ankle and/or elastic spasticity (Shefner et al., 2020).

### 2.3 Electrophysiological protocol

A standard EDX study was performed for all patients, including nerve conduction study (NCS) testing of at least 4 motor and 3 sensory nerves, and needle electromyography (EMG) of at least 2 muscles in the lower limbs, 2 muscles in the upper limbs, 1 muscle in the thoracic region, and 1 muscle in the bulbar region. All blink reflex recordings were performed by trained neurologists (JT, AP, EB, MM, or FB) in a quiet room with the temperature kept between 20 and 25 °C. Subjects were tested in the supine position and were asked to keep their eyes open. The blink reflex was elicited transcutaneously by electrical stimulation of the supraorbital nerve, with the cathode positioned at the supraorbital foramen and the anode 3 cm above it and 2 cm from the midline. The stimuli consisted of rectangular pulses of 1 ms duration and 15 mA intensity, delivered randomly to avoid habituation. Four stimuli were applied on each side. The responses were recorded from the orbicularis oculi muscles by surface electrodes, with active electrodes fixed to the lower eyelid and reference electrodes fixed to the temples (Supplementary Fig. 1). The EMG signals were fed into amplifiers (bandwidth 20 Hz-3 kHz) and stored on a local network. Latencies and amplitudes were measured on superimposed responses using cursors. The shortest latency for R1, R1′, R2, and R2′ responses was considered. Amplitude was measured from the onset latency marker to the peak of the negative phase, selecting the wave with the highest amplitude, for each response. An R1′ was considered if the amplitude of the corresponding negative wave was greater than 20 µV. A Nicolet® Viking® EDX (Natus Medical Incorporated, Middleton, WI, USA) was used.

### 2.4 Statistical analyses

Quantitative variables were described using median and interquartile ranges (IQR), and qualitative variables were described using frequencies. Proportions between groups were compared using the Chi-squared test with Yates’ continuity correction if the conditions were met, or with the non-parametric Fischer’s test otherwise.

The primary outcome was the presence of at least one R1′ in a subject. The comparisons between ALS patients and the healthy and non-ALS groups were performed sequentially in order to account for an overall alpha risk of 5 % (Chen and Wang, 2002). The comparison between ALS and non-ALS groups was performed if the first one was significant with an alpha risk of 5 %. The association was estimated using Odds Ratios (OR). The sensitivity and specificity for ALS of at least one R1′ and bilateral R1′ were calculated using non-SLA patients and healthy volunteers as non-affected individuals.

The sample size was calculated before the study to detect a frequency of R1′ of at least 28 % in the ALS group with an alpha risk of 5 % and a power of 80 % and estimated at 40 individuals in each group (ALS, non-ALS, and healthy volunteers), based on a prevalence of 5 % for R1′ in the general population according to previously published studies (Leon-S et al., 1995, Nacimiento et al., 1992).

As secondary outcomes were explored the amplitude and latency for other responses (R1, R2, and contralateral R2, hereafter referred to as R2′). Frequency of R1′ among ALS patients according to their bulbar or pseudobulbar characteristics was also explored.

Statistical analyses were conducted using R version 4.2.1 (R Core Team (2022), n.d.).

## 3 Results

### 3.1 Study population

A total of 120 participants were included in the study: 40 in the ALS group, 40 in the non-ALS group, and 40 in the healthy volunteer group. Among these participants, 58 were female (48.3 %). The median age at inclusion was 54 years (IQR: 41.0–66.0). A greater proportion of females was included in the healthy volunteer group (65.0 %) compared to the ALS and non-ALS groups (both 40.0 %), and these were younger (median age 40 years; IQR: 30.8–50.0) compared to those in the non-ALS group (median age 59 years; IQR: 52.0–74.3) and the ALS group (median age 60 years; IQR: 54.0–67.3). The median time from the onset of symptoms to inclusion was 11 months for ALS patients (IQR: 6.8–16.5) and 7 months for non-ALS patients (IQR: 3.3–22.5; Table 1). The most common diagnoses in the non-ALS group were autoimmune myasthenia gravis (22.5 %), functional neurological disorders (17.5 %), benign fasciculations (10.0 %), myositis (10.0 %), and radiculopathy (10.0 %; Table 2). The demographic characteristics, comorbidities, and medications of all included patients are presented in Supplementary Table 1.

**Table 1:** Epidemiological characteristics of patients according to the subgroup.

	**ALS (n = 40)**	**Non-ALS (n = 40)**	**Healthy volunteers (n = 40)**
Women, n (%)	16 (40.0 %)	16 (40.0 %)	26 (65.0 %)
Age at inclusion, years, median (IQR)	60.0 (54.0–67.3)	59.0 (52.0–74.3)	40.0 (30.8–50.0)
Time between first symptoms and inclusion, months (IQR)	11.0 (6.8–16.5)	7.0 (3.3–22.5)	/

ALS: amyotrophic lateral sclerosis, n: number, IQR: interquartile range.

**Table 2:** Diagnoses in the non-ALS group.

	**Non-ALS patients (n = 40)**
Auto-immune myasthenia gravis, n (%)	9 (22.5)
Functional neurologic disorder, n (%)	7 (17.5)
Benign fasciculation syndrome, n (%)	4 (10.0)
Myositis, n (%)	4 (10.0)
Radiculopathy/Lumbar spinal stenosis, n (%)	4 (10.0)
Genetic myopathies, n (%)	3 (7.5)
Distal hereditary motor neuropathy, n (%)	1 (2.5)
Dysphonia due to nasal septum deviation, n (%)	1 (2.5)
Isaacs syndrome with anti-CASPR2 antibodies, n (%)	1 (2.5)
Facial dystonia, n (%)	1 (2.5)
Multifocal motor neuropathy, n (%)	1 (2.5)
Nitrous oxide intoxication, n (%)	1 (2.5)
Neuralgic amyotrophy, n (%)	1 (2.5)
Cachexia, n (%)	1 (2.5)
Anti-Iglon5 disease, n (%)	1 (2.5)

ALS: amyotrophic lateral sclerosis, CASPR2: contactin-2 associated protein

### 3.2 Clinical characteristics of ALS patients

At inclusion, 39 ALS patients (97.5 %) had spinal involvement, 19 (47.5 %) had bulbar symptoms, 7 (17.5 %) had pseudobulbar affect, and 3 (7.5 %) had respiratory symptoms. Pyramidal syndrome was observed in 38 patients (95.0 %). The median ALSFRS-R score at inclusion was 42.0 (IQR: 39.8–44.3), and the median CNS-LS score was 7.0 (IQR: 7.0–8.5). Cognitive impairment was identified in five patients.

### 3.3 Blink reflex results

**Fig. 1:**
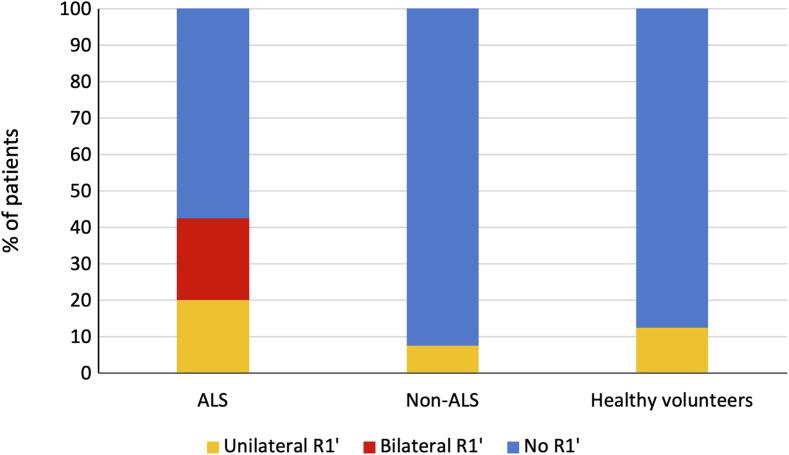
Proportion of patients with contralateral R1 responses (R1′) in each group. ALS: amyotrophic lateral sclerosis.

**Fig. 2:**
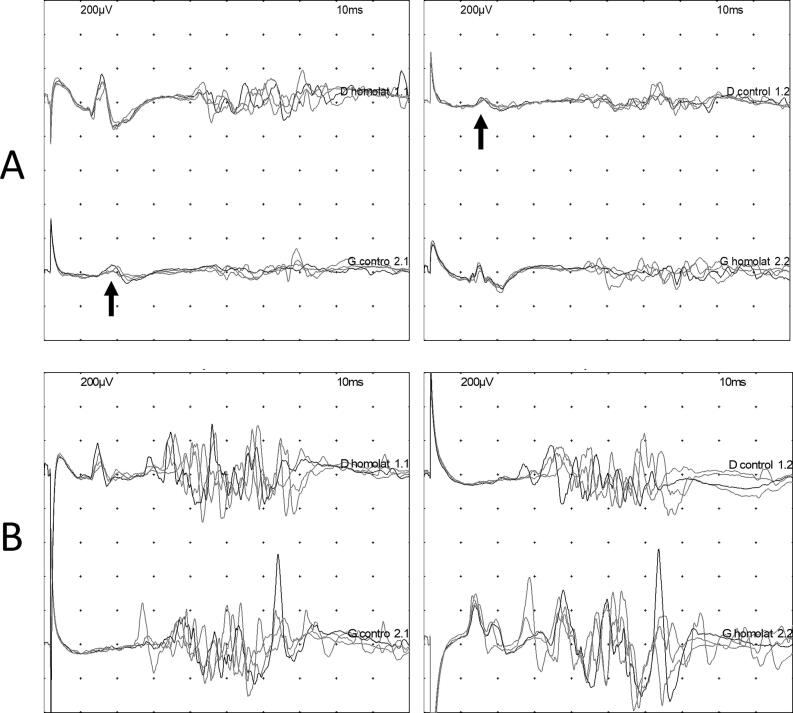
Illustrations of blink reflex in ALS and non-ALS patients. (A) ALS patient with bilateral contralateral R1 responses (R1′), with an amplitude of 45.4 µV after right stimulation (top line) and 64.3 µV after left stimulation (bottom line). (B) Non-ALS patient (radiculopathy) without R1′. For each patient, right stimulation is presented on the top line, with ipsilateral responses (“D homolat”) on the left and contralateral responses (“D control”) on the right. Left stimulation is presented on the bottom line, with ipsilateral responses on the right (“G homolat”) and contralateral responses on the left (“G control”). Black arrow: R1’.

Regarding the primary outcome of this study, the proportion of individuals with at least one R1′ was significantly higher in ALS patients (42.5 %) compared to healthy volunteers (12.5 %, OR = 4.98, p-value = 0.00588) and non-ALS patients (7.5 %, OR = 8.56, p-value = 0.000789; Table 3, Fig. 1). The three non-ALS patients with R1′ were diagnosed with inclusion body myositis, distal hereditary motor neuropathy, and benign fasciculation syndrome. Regarding the presence of at least one R1′, the sensitivity was 42.5 % and the specificity 90.0 %. Bilateral R1′ (i.e. after right and left stimulations) was observed only in the ALS group (9 patients, 22.5 %; Table 3, Fig. 1). The sensitivity of bilateral R1′ was 22.5 %, and the specificity was 100.0 %. Illustrations of blink reflex results for a patient with ALS (A) and a non-ALS patient with radiculopathy (B) are presented in Fig. 2.

**Table 3:** Electrophysiological results of the blink reflex in the three groups.

	**ALS (n = 40)**	**Non-ALS (n = 40)**	**Healthy volunteers (n = 40)**
Number of patients with at least one R1′ (%)	17 (42.5)	3 (7.5 %)	5 (12.5 %)
Number of patients with bilateral R1′ (%)	9 (22.5 %)	0 (0.0 %)	0 (0.0 %)
Median latency of R1′, ms (IQR)	13.7 (12.8–14.5)	11.9 (11.2–12.9)	11.5 (11.3–12.2)
Median amplitude of R1′, μV (IQR)	45.3 (34.2–64.2)	52.0 (39.4–52.1)	29.2 (29.0–43.9)
Median latency of R1, ms (IQR)	12.1 (11.2–12.6)	11.9 (11.4–12.6)	12.2 (11.5–13.0)
Median amplitude of R1, μV (IQR)	185.7 (122.5–324.54)	161.1 (121.2–230.2)	188.7 (144.4–357.3)
Median latency of R2, ms (IQR)	34.6 (31.7–37.4)	32.5 (29.5–35.6)	32.0 (30.0–34.1)
Median amplitude of R2, μV (IQR)	172.0 (122.0–321.1)	219.18 (152.3–313.2)	290.1 (151.9–356.1)
Median latency of R2′, ms (IQR)	32.3 (30.5–36.9)	32.0 (29.5–35.7)	30.15 (28.6–33.3)
Median amplitude of R2′, μV (IQR)	171.8 (110.9–266.2)	196.2 (117.9–274.1)	211.7 (175.6–277.7)

ALS: amyotrophic lateral sclerosis,’: contralateral responses, n: number, IQR: interquartile range.

As for the secondary outcomes, there was no clinically significant difference in the latencies and amplitudes of R1′ (as well as R1, R2, and R2′) between the groups (Table 3).

Among ALS patients, those with bulbar symptoms did not have significantly more R1′ (n = 9/19, 47.4 %) than those without (n = 8/21, 38.1 %, p = 0.7855). There was a trend towards more R1′ among ALS patients with pseudobulbar (n = 5/7, 71.4 %) than those without (n = 12/33, 36.4 %, p = 0.1134). Among the five ALS patients with cognitive impairment, four had at least one R1′. The median time from the onset of symptoms to the EDX study did not differ between ALS patients with R1′ (12 months, IQR: 8.0–18.0) and those without R1′ (11 months, IQR: 6.5–13.0; p-value = 0.4506).

## 4 Discussion

In the present study, the frequency of the R1′ in the blink reflex in patients with ALS was compared to that found in healthy volunteers and patients with motor deficits and/or fasciculations of other etiology. A higher frequency of R1′ in the ALS group was observed, which was approximately four times higher than in the other two groups.

Bilateral R1′ was found only in the ALS group, which was specific but not sensitive because observed in under a quarter of the cases. It therefore seems that a R1′ observed during both right and left stimulations may support the diagnosis of ALS. However, for R1′ observed only unilaterally, the diagnostic value appears less evident because of a lower specificity as this type of response was observed in healthy volunteers as well as in non-ALS patients. For non-ALS patients, a unilateral R1′ was, for example, observed in a patient with benign fasciculations, which represents a key differential diagnosis for ALS. For healthy subjects, it is known that R1′ can be rarely found, particularly after facilitating maneuvers such as a gentle contraction of the orbicularis oculi and conditioning stimulus of the median nerve (Soliven et al., 1988, Willer et al., 1984). However, this response is absent in the majority of healthy subjects (Leon-S et al., 1995, Nacimiento et al., 1992). Regarding sensitivity, most ALS patients did not exhibit an R1′, which does not make it a sensitive marker. The amplitude of the R1′ was low compared with the R1 response in all three groups, which could limit its usefulness in clinical practice, and this response should be considered only if reproducible after repeated stimulations.

Regarding the latencies and amplitudes of the other responses of the blink reflex, no clinically significant difference was found between the three groups. In a previous study, the latencies of these responses were also similar between ALS patients and healthy controls; moreover, an alteration of the blink reflex recovery cycle (BRRC) was found, also suggesting brainstem hyperexcitability (Cengiz et al., 2017). However, in four other studies, low amplitude and/or prolonged latency of R2 and R2′ responses were observed in ALS patients compared to controls (Podikoglou, 2004, Rostás et al., 2023, Shimoda et al., 2009, Szmidt-Sałkowska and Rowińska-Marcińska, 2005). This difference in findings regarding the R2 and R2′ responses seems not to be explained by the interval between the first symptoms and the electrophysiological study, since this was shorter in certain previously published studies than herein (Podikoglou, 2004, Rostás et al., 2023).

The results of this study are of interest from a pathophysiological perspective. As suggested in patients with HTLV1-associated encephalopathy, the appearance of an R1′ to the blink reflex could reflect the loss of corticobulbar neurons from the motor cortex to the brainstem nuclei, and their inhibitory role on secondary motor neurons (Leon-S et al., 1995). This is supported herein by the trend towards a higher frequency of R1′ among patients with a pseudobulbar affect, and the absence of a trend towards a higher frequency of R1′ among those with bulbar symptoms. In other words, R1′ could be the electrophysiological equivalent of the pseudobulbar affect observed in ALS patients and characterized by uncontrolled episodes of crying and laughing (Nabizadeh et al., 2022, Nguyen and Matsumoto, 2019). It would be interesting, in a dedicated study, to investigate the presence of an R1′ among patients with a pseudobulbar affect, and to evaluate modulation of this response by serotoninergic drugs or the dextromethorphan/quinidine association, usually prescribed to treat this symptom; in addition, the correlation of R1′ with other structural markers of corticobulbar dysfunction could be investigated using, for example, diffusion tensor imaging.

The present study has limitations. It is a single-center study, and the results will need to be reproduced in other centers for a more robust validation. All the patients included had motor deficits of peripheral origin in the non-ALS group. However, it would be interesting to investigate the frequency of the R1′ in other central pathologies, such as progressive supranuclear palsy (PSP), which can mimic a bulbar form of ALS and has been associated with brainstem hyperexcitability as shown by altered BRRC (Valls-Sole, 1997). The final limitation is the strict definition of pseudobulbar symptoms as a CNS-LS score > 12, which excludes patients with pseudobulbar involvement who do not present with pseudobulbar affect.

In conclusion, the present study found a higher frequency of R1′ in ALS patients compared to controls, and bilateral R1′ was specific to ALS patients. Thus, an R1′, especially when bilateral, could be useful in the setting of differential diagnosis of ALS, as a marker of corticobulbar dysfunction. It is nonetheless essential to integrate this finding into the wider clinical context.

## Declaration of competing interest

The authors declare that they have no known competing financial interests or personal relationships that could have appeared to influence the work reported in this paper.
