# Supplementary material for: Contralateral R1 response in blink reflex in patients with amyotrophic lateral sclerosis
Source: Clin Neurophysiol Pract. 2025 Feb 22;10:47–51. doi: 10.1016/j.cnp.2025.02.005 (PMC11909417; doi:10.1016/j.cnp.2025.02.005)
Supplement: Supplementary Data 1 [file mmc1.docx]

**Supplementary Table 1**: Demographic characteristics, comorbidities, and medications of ALS and non-ALS patients.

| **Patient** | **Group** | **Sex** | **Diagnosis in non-ALS group** | **Age at inclusion, years** | **Time between first symptoms and inclusion, months** | **Comorbidities** | **Medications** |
| --- | --- | --- | --- | --- | --- | --- | --- |
| 1 | ALS | F | / | 47 | 10 | None | None |
| 2 | ALS | M | / | 60 | 4 | Migraine, Glaucoma | Brinzolamide, Tafluprost/Timolol |
| 3 | ALS | M | / | 55 | 24 | Epilepsy | Depakine, Tegretol |
| 4 | ALS | F | / | 76 | 48 | Lupus, CRPS | Pregabalin, Melatonin, Baclofen |
| 5 | ALS | F | / | 51 | 8 | Psoriasis, Urticaria, Depression, Hypothyroidism, Cervicobrachial Neuralgia | Levothyroxine, Cetirizine, Venlafaxine, Diazepam, Baclofen, Gabapentin, Paracetamol |
| 6 | ALS | F | / | 79 | 10 | Hypertension, Rheumatoid Arthritis | Methotrexate, Vitamin B9, Hydrochlorothiazide, Irbesartan |
| 7 | ALS | F | / | 66 | 5 | Hypothyroidism, Osteoarthritis, Scoliosis | Levothyroxine |
| 8 | ALS | F | / | 80 | 26 | Rheumatoid Arthritis | Methotrexate, Vitamin B9 |
| 9 | ALS | M | / | 67 | 9 | Depression | Paroxetine |
| 10 | ALS | F | / | 67 | 11 | Depression, Mitral Insufficiency, Pancreatic Neuroendocrine Tumor | Venlafaxine, Pancreatin |
| 11 | ALS | F | / | 40 | 7 | Migraine | Triptan |
| 12 | ALS | F | / | 55 | 9 | Chronic Veinous Insufficiency, Hypothyroidism | Levothyroxine |
| 13 | ALS | M | / | 54 | 12 | Hypertension | Nebivolol, Hydrochlorothiazide, Amlodipine, Perindopril |
| 14 | ALS | M | / | 62 | 11 | Hypercholesterolemia, Hypothyroidism | Levothyroxine |
| 15 | ALS | M | / | 36 | 8 | None | None |
| 16 | ALS | F | / | 90 | 16 | Hypertension | Acebutolol |
| 17 | ALS | M | / | 66 | 48 | COPD, Prostate Cancer, Hypercholesterolemia | Pravastatin |
| 18 | ALS | F | / | 76 | 14 | Pruritus Sine Materia | Paracetamol |
| 19 | ALS | F | / | 49 | 8 | None | None |
| 20 | ALS | F | / | 61 | 24 | GERD, Depression | Sertraline |
| 21 | ALS | M | / | 54 | 3 | None | None |
| 22 | ALS | M | / | 57 | 4 | Osteoarthritis | None |
| 23 | ALS | M | / | 72 | 12 | None | None |
| 24 | ALS | F | / | 57 | 15 | Obesity, Hypertension, Trigeminal neuralgia | Tegretol, Hydrochlorothiazide, Irbesartan |
| 25 | ALS | M | / | 46 | 22 | None | None |
| 26 | ALS | M | / | 82 | 18 | Hypertension, Type 2 Diabetes | Insulin, Metformin, Amlodipine, Perindopril |
| 27 | ALS | M | / | 58 | 20 | Asthma | None |
| 28 | ALS | F | / | 78 | 12 | Hypertension, Carpal Tunnel Syndrome | Spironolactone |
| 29 | ALS | M | / | 63 | 18 | None | None |
| 30 | ALS | M | / | 55 | 6 | Type 2 Diabetes, Hepatic Steatosis | Metformin |
| 31 | ALS | M | / | 60 | 18 | None | None |
| 32 | ALS | M | / | 68 | 5 | Lumbar Spinal Stenosis, Prostate Cancer, Depression | Paroxetine |
| 33 | ALS | M | / | 52 | 12 | Hypertension, Hypercholesterolemia | None |
| 34 | ALS | M | / | 54 | 4 | Hypercholesterolemia | None |
| 35 | ALS | M | / | 83 | 4 | Hypertension, Prostate Cancer, COPD | Silodosin, Vilanterol/Umeclidinium |
| 36 | ALS | M | / | 57 | 14 | None | None |
| 37 | ALS | M | / | 66 | 7 | Carpal Tunnel Syndrome | None |
| 38 | ALS | M | / | 61 | 5 | None | None |
| 39 | ALS | F | / | 51 | 4 | None | None |
| 40 | ALS | M | / | 66 | 12 | Hypertension, Lumbar Spinal Stenosis | Amlodipine, Bisoprolol |
| 41 | Non-ALS | M | Myositis | 86 | 24 | Hypertension, Type 2 Diabetes, Hypercholesterolemia, Rheumatoid Arthritis | Atenolol, Furosemide, Metformin, Hydroxyurea, Atorvastatin |
| 42 | Non-ALS | M | dHMN | 52 | 36 | None | None |
| 43 | Non-ALS | M | Isaacs syndrome with anti-CASPR2 antibodies | 85 | 2 | Hypertension, Hypercholesterolemia, Type 2 Diabetes, Auto-immune Myasthenia gravis, Atrial Fibrillation | Ambenonium, Rivaroxaban, Perindopril, Amlodipine |
| 44 | Non-ALS | F | Auto-immune myasthenia gravis | 88 | 7 | Hypertension | Irbesartan, Hydrochlorothiazide |
| 45 | Non-ALS | M | Benign fasciculation syndrome | 40 | 8 | None | None |
| 46 | Non-ALS | F | Functional neurologic disorder | 58 | 3 | None | None |
| 47 | Non-ALS | M | Functional neurologic disorder | 67 | 120 | Benign Prostatic Hyperplasia | Non |
| 48 | Non-ALS | M | Benign fasciculation syndrome | 74 | 36 | Asthma, Meniere's Disease, Hypercholesterolemia | Salbutamol |
| 49 | Non-ALS | M | Dysphonia due to nasal septum deviation | 61 | 5 | Hypertension | Nebivolol, Aspirin, Perindopril |
| 50 | Non-ALS | M | Genetic myopathy | 56 | 12 | GERD, Chronic Veinous Insufficiency | Omeprazole |
| 51 | Non-ALS | F | Auto-immune myasthenia gravis | 75 | 6 | Hypertension, Hypothyroidism, OSAS | Levothyroxine, Perindopril |
| 52 | Non-ALS | F | Facial dystonia | 46 | NA | None | None |
| 53 | Non-ALS | M | Auto-immune myasthenia gravis | 59 | 1 | Hypertension, Type 1 Diabetes, Biermer's Disease | Amlodipine, Metformin, Vitamin B12, Pantoprazole |
| 54 | Non-ALS | M | Multifocal motor neuropathy | 76 | 4 | Depression, Hypercholesterolemia | Paroxetin |
| 55 | Non-ALS | F | Auto-immune myasthenia gravis | 59 | 2 | Obesity, Canal Tunnel Syndrome | None |
| 56 | Non-ALS | F | Functional neurologic disorder | 54 | 7 | Depression | None |
| 57 | Non-ALS | F | Nitrous oxide intoxication | 25 | 12 | None | None |
| 58 | Non-ALS | M | Radiculopathy | 66 | 5 | Rhizomelic Pseudopolyarthritis | None |
| 59 | Non-ALS | F | Auto-immune myasthenia gravis | 57 | 1 | Depression | Venlafaxine |
| 60 | Non-ALS | M | Neuralgic amyotrophy | 71 | 5 | Hypertension, COPD | Amlodipine, Bisoprolol, Tiotropium |
| 61 | Non-ALS | M | Cachexia | 88 | NA | Atrial Fibrillation, COPD, GERD | Apixaban, Pantoprazole, Ipratropium, Budesonide/Formoterol |
| 62 | Non-ALS | M | Functional neurologic disorder | 21 | 1 | None | None |
| 63 | Non-ALS | F | Myositis | 67 | 12 | GERD, COPD | Omeprazole, Budesonide/Formoterol |
| 64 | Non-ALS | M | Benign fasciculation syndrome | 62 | 12 | Migraine, Carpal Tunnel Syndrome | Non |
| 65 | Non-ALS | F | Myositis | 52 | 3 | Hypertension, lower limbs radiculopathy | Perindopril, Aspirin |
| 66 | Non-ALS | F | Auto-immune myasthenia gravis | 47 | 1 | Psoriasis | None |
| 67 | Non-ALS | M | Functional neurologic disorder | 53 | 18 | Lumbar Spinal Stenosis | None |
| 68 | Non-ALS | M | Benign fasciculation syndrome | 35 | 6 | None | None |
| 69 | Non-ALS | F | Auto-immune myasthenia gravis | 85 | 9 | Depression | Mirtazapine, Oxazepam |
| 70 | Non-ALS | M | Radiculopathy | 54 | 3 | Chronic Alcohol Abuse | Vitamin B1, Vitamin B6 |
| 71 | Non-ALS | F | Genetic myopathy | 52 | 2 | None | None |
| 72 | Non-ALS | F | Myositis | 75 | 48 | Peripheral Arterial Disease | Aspirin, Perindopril, Simvastatin |
| 73 | Non-ALS | M | Functional neurologic disorder | 55 | 24 | Fibromyalgia | Amitriptyline |
| 74 | Non-ALS | M | Functional neurologic disorder | 51 | 24 | Hyperthyroidism, Essential Tremor | Propranolol |
| 75 | Non-ALS | F | Auto-immune myasthenia gravis | 72 | 6 | Obesity, Type 2 Diabetes, Hypertension, Rheumatoid Arthritis | Metformin, Esomeprazole, Valsartan, Amlodipine |
| 76 | Non-ALS | M | Lumbar spinal stenosis | 79 | 36 | Prostate Cancer | Pregabalin |
| 77 | Non-ALS | M | Genetic myopathy | 39 | 120 | Cardiomyopathy | Apixaban |
| 78 | Non-ALS | M | Anti-Iglon5 disease | 70 | 36 | Prostate Cancer, OSAS, Depression | Sertraline |
| 79 | Non-ALS | M | Lumbar spinal stenosis | 87 | 7 | Peripheral Arterial Disease, Prostate Cancer | Aspirin, Perindopril, Simvastatin |
| 80 | Non-ALS | F | Auto-immune myasthenia gravis | 42 | 12 | Obesity | None |

ALS: amyotrophic lateral sclerosis, CASPR2: contactin-2 associated protein CRPS: complex regional pain syndrome, COPD: chronic obstructive pulmonary disease, dHMN: distal hereditary motor neuropathy GERD: gastroesophageal reflux disease, F: female, M: male, NA: not available, OSAS: obstructive sleep apnea syndrome
