# Supplementary material for: Contralateral R1 response in blink reflex in patients with amyotrophic lateral sclerosis
Source: Clin Neurophysiol Pract. 2025 Feb 22;10:47–51. doi: 10.1016/j.cnp.2025.02.005 (PMC11909417; doi:10.1016/j.cnp.2025.02.005)
Supplement: Supplementary Data 2 [file mmc2.docx]

**
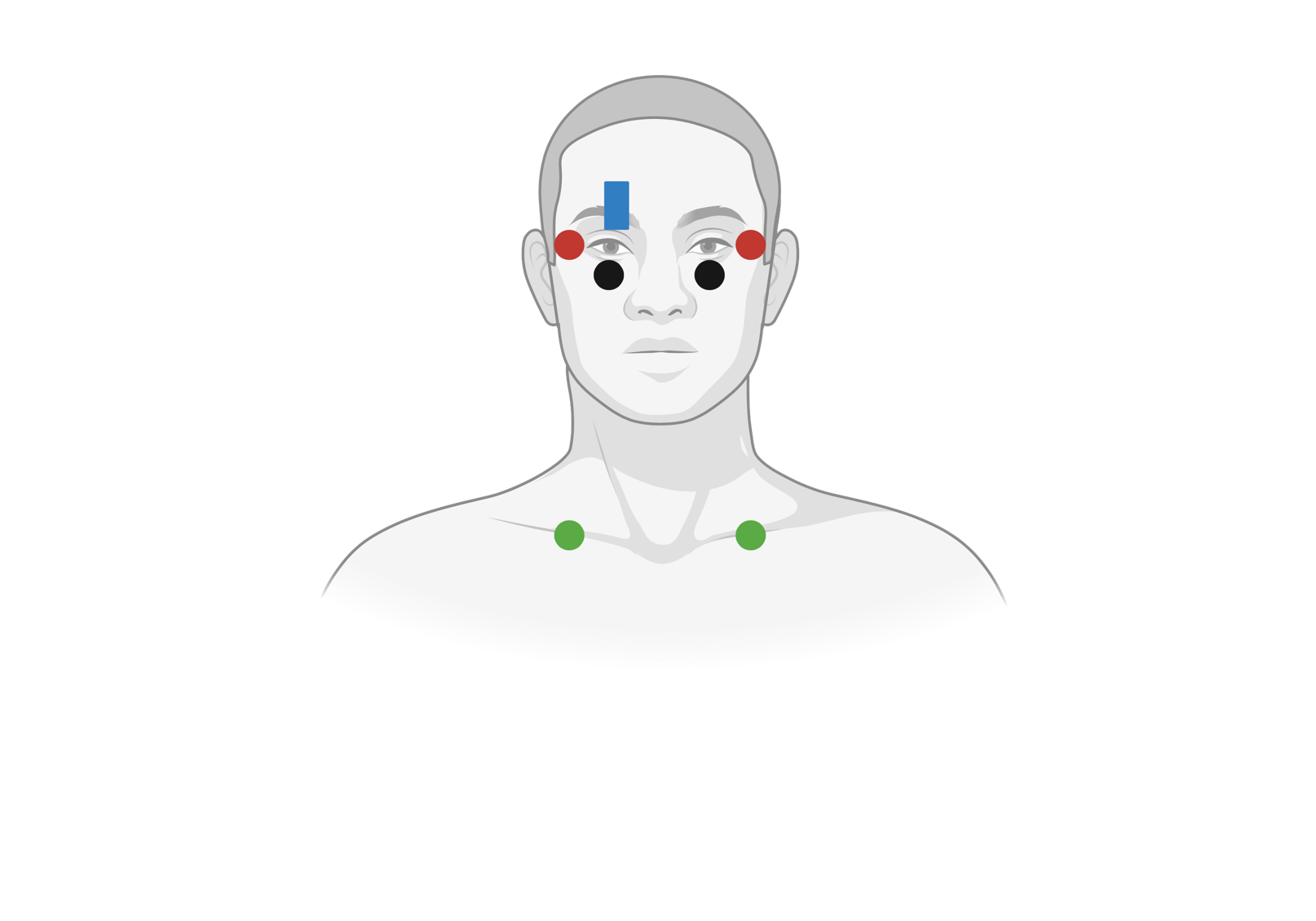
Supplementary Fig. 1.** Position of electrodes during blink reflex recording.

Black circles: active electrodes, red circles: reference electrodes, green circles: ground electrodes, blue rectangle: stimulator. Designed with BioRender.com.
